# Caraway Essential Oil Nanoparticles in Prolonged Stability and Sensory Improvement of Fresh Pork Sausages

**DOI:** 10.3390/foods15091591

**Published:** 2026-05-04

**Authors:** Nenad Jevremović, Božana Odžaković, Natalija Đorđević, Dani Dordevic, Ivica Zdravković, Ivana Karabegović, Bojana Danilović

**Affiliations:** 1 Rubin A.D., Cara Lazara 291, 37000 Kruševac, Serbia; nenad.jevremovic@rubin.rs; 2 Faculty of Technology, University of Banja Luka, Bulevar Vojvode Stepe Stepanovića 73, 78000 Banja Luka, Bosnia and Herzegovina; bozana.odzakovic@tf.unibl.org; 3 Faculty of Technology, University of Niš, Bulevar Oslobođenja 124, 16000 Leskovac, Serbia; natalija@tf.ni.ac.rs (N.Đ.); ivana.karabegovic@tf.ni.ac.rs (I.K.); 4 Department of Plant Origin Food Sciences, Faculty of Veterinary Hygiene and Ecology, University of Veterinary Sciences, 61242 Brno, Czech Republic; dordevicd@vfu.cz; 5 Department of Leskovac Business School, Academy of Vocational Studies of Southern Serbia, Vlade Jovanovića 8, 16000 Leskovac, Serbia; zdravkovic.ivica@vpsle.edu.rs

**Keywords:** caraway essential oil, chitosan nanoparticles, nanoencapsulation, fresh pork sausages, antimicrobial activity, antioxidant activity, shelf life extension

## Abstract

Caraway essential oil (CEO) and chitosan-based nanoparticles incorporating CEO (CNPs CEO) were evaluated as natural preservatives for fresh pork sausages stored at +4 °C for five days. The chemical composition of CEO was characterized by gas chromatography–mass spectrometry (GC/MS) and gas chromatography with flame ionization detection (GC/FID); carvone (92.5%) and limonene (5.8%) were identified as dominant components. Eight experimental treatments were applied: control, CEO at 0.2, 0.4, and 0.6 mg/g, chitosan nanoparticles (CNPs), and CNPs CEO at 0.2, 0.4, and 0.6 mg/g. Encapsulation efficiency of CEO in chitosan nanoparticles was 67.7 ± 1.91%. Microbiological quality (total bacterial count (TBC), lactic acid bacteria, yeasts and moulds), lipid oxidation (TBARS), pH, and sensory attributes of raw and thermally processed sausages were monitored throughout storage. CEO reduced microbial growth and lipid oxidation in a concentration-dependent manner, while CNPs CEO formulations showed markedly superior performance. The CNPs CEO 0.6 mg/g treatment achieved the greatest inhibitory effect on all microbiological parameters, reducing TBC for 1.6 log CFU/g and limiting lipid oxidation, yielding final malondialdehyde values of 1.15 mg MDA/kg, approximately 50% lower than the control (2.18 mg MDA/kg). Sensory evaluation indicated that CNPs CEO-treated sausages maintained acceptable colour, odour, juiciness, texture, and overall acceptability throughout the storage period. The sample treated with CNPs CEO 0.6 mg/g remained above the acceptability level for all analyzed parameters for 5 days of storage, while the control became unacceptable for lipid oxidation on the fifth day and sensory unacceptable after the third day. These findings demonstrate that the application of CNPs CEO in sausage production enhances their stability, shelf life, and sensory characteristics, indicating a promising no-additive strategy in the industrial production of fresh pork sausages.

## 1. Introduction

Sausages are meat products derived from fresh, raw minced meat of various animal species, including pork, chicken, beef, and fish, and are regarded as some of the most widely consumed and nutritionally valuable meat products. During the production of raw pork sausages, a variety of spices, including salt, pepper, paprika, caraway, oregano, and others, are incorporated into the minced meat, depending on the specific formulation used by the manufacturer [1]. Fresh sausages are classified as highly perishable food products, as they are produced from raw ground meat that provides a favourable environment for the growth and proliferation of microorganisms [2]. Microorganisms frequently present on raw meat and meat products can significantly affect their sensory and biochemical characteristics, and their consumption may lead to the transmission of foodborne illnesses [3]. Owing to their high fat content, raw pork sausages are also particularly susceptible to lipid oxidation, necessitating storage in appropriate packaging under cool and dark conditions [4]. Sausage spoilage can result from microorganisms and/or enzymes present in the meat, which trigger biochemical reactions. These biochemical processes progressively accelerate until the complete degradation of sensitive compounds [2].

In the food industry, synthetic preservatives are widely used to inhibit microbial proliferation and slow down lipid oxidation. However, due to economic considerations and growing consumer concerns about the safety of foods containing synthetic additives, significant attention has shifted toward natural bioactive compounds [5]. Essential oils represent a significant group of natural products with potential for use in food preservation. These secondary metabolites, synthesized by aromatic and medicinal plants, contain volatile molecules responsible for their characteristic aroma and flavour. The bioactive constituents of essential oils, such as terpenes and hydrocarbons, are well known for their antimicrobial, antifungal, antiviral, antimycotic, antiparasitic, insecticidal, and antioxidant properties [6].

Caraway essential oil (CEO), due to its content of aromatic and bioactive compounds, is widely applied in the food and pharmaceutical industries. It has been shown to effectively inhibit the growth of various Gram-positive and Gram-negative bacteria, including *Bacillus cereus*, *Bacillus subtilis*, *Staphylococcus aureus*, *Salmonella typhi*, and *Listeria innocua*, as well as fungi *Candida albicans*, *Aspergillus niger*, and others [5,7]. Carvone and limonene, which are the predominant constituents of its chemical composition, are considered the main compounds responsible for the inhibition of bacterial and fungal growth [8,9,10].

Nanoencapsulation has been widely investigated as an effective strategy to overcome the technological limitations associated with the direct application of essential oils in food systems, including high volatility, susceptibility to oxidation, low water solubility and rapid loss of biological activity during processing and storage [11,12]. This approach involves the entrapment of essential oil components within nanometre-sized carriers composed of natural or synthetic matrices, which physically isolate bioactive compounds from external environmental factors such as oxygen, light and heat, while reducing their undesirable interactions with food constituents. As a result, nanoencapsulation significantly enhances the physicochemical stability of essential oils and allows their gradual and controlled release, thereby prolonging their antimicrobial and antioxidant effectiveness compared to non-encapsulated forms [11,13].

In food preservation applications, nanoencapsulated essential oils have demonstrated improved functionality due to their increased surface-to-volume ratio, enhanced dispersion in aqueous food matrices and more efficient interaction with microbial cells. Chitosan is a particularly attractive carrier matrix owing to its intrinsic antimicrobial properties, biocompatibility, biodegradability, and capacity for ionic gelation with tripolyphosphate to form stable nanoparticles [14,15,16]. The controlled release behaviour of nanoencapsulation systems enables sustained antimicrobial and antioxidant activity over time, which is particularly relevant for perishable food products, including meat and meat-based products, where microbial growth and lipid oxidation are the primary causes of quality deterioration [12,13]. Moreover, nanoencapsulation has been reported to reduce the strong aroma and flavour impact of essential oils, allowing their application at lower effective concentrations without negatively affecting the sensory properties of food products [11,12]. Consequently, nanoencapsulation represents a promising technological tool for the development of natural preservation strategies aimed at extending shelf life and reducing reliance on synthetic additives in food systems.

Despite the growing literature on essential oil nanoencapsulation in meat systems, several important gaps remain unaddressed. The majority of published studies have focused on beef or poultry, sheep or beef matrices [15,17,18,19,20,21,22,23], with comparatively limited investigation of fresh pork sausages, which present distinct challenges due to their high fat content, minced texture, mixed spice background, and susceptibility to rapid deterioration during refrigerated storage. Furthermore, while caraway essential oil has been examined in fermented and dry-cured sausage formulations [24,25], its application in fresh pork sausage systems where the full complexity of raw meat, fat, and added spices simultaneously interacts with the preservative has received only limited systematic evaluation. Moreover, the comparative performance of free CEO compared to chitosan-nanoencapsulated CEO across quality parameters (microbiological, oxidative, pH, and sensory) within a single experimental design has not been documented for this product category.

The aim of the present study is to address these gaps by evaluating the effect of free CEO and chitosan-nanoparticle-incorporated CEO at three concentration levels on the microbiological quality, lipid oxidation, pH stability, and sensory attributes of fresh pork sausages stored under refrigeration for five days. By directly comparing free and encapsulated forms under identical food-system conditions, this work provides quantitative evidence for the added value of nanoencapsulation and contributes to the scientific foundation needed to support the development of clean-label preservation strategies for the fresh sausage industry.

## 2. Materials and Methods

### 2.1. Hydrodistillation of Caraway Essential Oil

The CEO was obtained by microwave-assisted hydrodistillation with a Clevenger apparatus from caraway seed purchased from a retailer. A mixture of caraway seeds and distilled water, in a 1:10 ratio, and microwave-assisted hydrodistillation was performed for 60 min, after which the obtained oil was separated and stored at +4 °C until use.

### 2.2. GC/MS and GC/FID Analysis of Caraway Essential Oil

GC/MS analysis was performed using a gas chromatograph (Agilent Technologies, 7890B Gas Chromatograph, Santa Clara, CA, USA) coupled with a selective mass detector (5977A MSD, Agilent Technologies, USA). The components were separated on a non-polar HP-5MS silica capillary column (stationary phase composed of 5% diphenyl and 95% dimethylpolysiloxane; 30 m length, 0.25 mm internal diameter, and 0.25 µm film thickness; Agilent Technologies, USA). The samples were dissolved in diethyl ether, and 1 μL of the prepared solution was injected into the GC column via a split/splitless inlet maintained at 220 °C and operated in 40:1 split mode. High-purity helium (99.999%) was used as the carrier gas at a constant flow rate of 1 cm^3^/min. The oven temperature was programmed to increase linearly from 60 °C to 246 °C at a rate of 3 °C/min. The total analysis time was 62 min. The eluate was transferred to the quadrupole mass spectrometer via a transfer line maintained at 250 °C and analyzed using electron ionization (EI) in Scan mode. The ion source and quadrupole analyser temperatures were set at 230 °C and 150 °C, respectively, with an electron energy of 70 eV. The mass spectral data were acquired over a scan range of *m*/*z* 41–415 Da. Further analysis was performed using gas chromatography with flame ionization detection (GC/FID) on the same instrument equipped with a post-column split unit, under identical chromatographic conditions as those applied for GC/MS analysis. The flow rates of the FID gases were as follows: carrier gas (He) 1 mL/min, make-up gas (N_2_) 25 mL/min, fuel gas (H_2_) 30 mL/min, and oxidizing gas (air) 400 mL/min. The FID temperature was maintained at 300 °C.

#### 2.2.1. Data Processing

Data processing was conducted using MSD ChemStation Data Analysis software (revision F.01.00.1903) in combination with AMDIS (Automatic Mass Spectral Deconvolution and Identification System, version 2.70) and NIST MS Search (version 2.0g) software (Agilent Technologies, USA). For unambiguous identification of the mixture components, experimentally determined retention indices were used, based on a homologous series of n-alkanes (C_8_–C_20_) as standards. Component identification was performed by comparing their experimental retention indices (RI^exp^) with values reported in the literature (RI^lit^) [26], by comparing their mass spectra with those from the Wiley, NIST, and RTLPEST libraries (MS), and, whenever possible, by co-injection of the corresponding reference standards (Co-I).

#### 2.2.2. Quantitative Analysis of Caraway Essential Oil

Quantitative analysis was performed using the external standard method, with standard solutions of limonene (0.5–4 mg/mL) and linalool (1.67–15 mg/mL) prepared in a defined concentration range. The mean response factor (RF_mean_) was used for the quantification of analytes in the sample [27]. The percentage concentration (C, %) of each component in the sample was calculated as the ratio of the individual component concentration to the total concentration of all components in the sample.

### 2.3. Preparation of Chitosan Nanoparticles with Incorporated Caraway Essential Oil

Chitosan-based nanoparticles with added CEO were prepared using the ionic gelation method [16]. A 0.4% chitosan solution was prepared by dissolving chitosan powder of medium molecular weight 190–310 kDa (Sigma-Aldrich, St. Louis, MO, USA) in a 1% acetic acid solution (>99.8%, Centrohem, Stara Pazova, Serbia), with constant stirring on a magnetic stirrer at room temperature (SCILOGEX SCI280-Pro, Rocky Hill, CT, USA). The resulting solution was filtered to remove undissolved particles, after which the pH of the filtrate was adjusted to 4.7–4.8, and 0.1% of the surfactant Tween 60 (Fisher Scientific, Loughborough, UK) was added. The solution was then stirred on a magnetic stirrer for 30 min at 60 °C until a homogeneous consistency was achieved. The CEO (200 μL) dissolved in 4 mL of ethanol (96%) was added to the chitosan solution dropwise, with constant stirring at 1200 rpm for 30 min. After homogenization at room temperature, 30 mL of sodium tripolyphosphate solution (>96%, Centrohem, Stara Pazova, Serbia) (1.87 mg/mL, pH = 4) was added dropwise, with gentle stirring for 60 min. The suspension of caraway essential oil-enriched nanoparticles (80 mL) was obtained by centrifugation of the resulting solution at 14,000 rpm for 20 min (Eppendorf 5418 Centrifuges, Eppendorf, Hamburg, Germany) and stored at +4 °C until use.

### 2.4. Determination of Encapsulation Efficiency

For the determination of encapsulation efficiency, 200 μL of CNPs CEO suspension in deionised water was added to 5 mL of a 2 mol/L HCl solution, followed by incubation in a boiling water bath for 30 min. After cooling, 2 mL of ethanol (96%) was added to the cooled system, and centrifugation was performed at 9000 rpm for 5 min. The absorbance spectrum of the supernatant was measured using a spectrophotometer (2100 UV Spectrophotometer, Cole-Parmer, Vernon Hills, IL, USA) in the wavelength range of 200 to 400 nm. Encapsulation efficiency was calculated using the following formula:

Encapsulation efficiency (%) = (mass of incorporated essential oil/mass of added essential oil) × 100

### 2.5. Preparation of Raw Sausage Samples

The control sample of raw sausage was prepared by mixing 7 kg of fresh pork shoulder meat and 3 kg of firm pork adipose tissue with 2% salt, 1% ground paprika, 0.3% black pepper, and 0.3% garlic. The meat and adipose tissue were minced and mixed with the seasonings in order to obtain the sausage dough. Based on this control mixture, seven additional samples were prepared by incorporating the following additives into the sausage dough. Specifically, CEO was added at concentrations of 0.2 mg/g, 0.4 mg/g, and 0.6 mg/g in the first set of samples. In the second set, CNPs were introduced, and in the third set, CNPs CEO in the form of suspension were added at concentrations of 0.2 mg/g, 0.4 mg/g, and 0.6 mg/g (Table 1). Equal distribution of the CEO and nanoparticles was obtained by mixing the mixture in the sausage mixer. The sausage mixture was then stuffed into artificial casings, twisted, and the raw sausage samples were packed in sterile bags (Whirl-Pak^®^ Sample Bag, 384 mL, Madison, WI, USA) and stored at +4 °C for 5 days. Each day, the raw sausages were analyzed for oxidative and microbial stability and sensory analysis, while samples from each batch of sausages were baked and screened for sensory analysis.

### 2.6. Determination of pH Value

To determine the pH value of fresh pork sausage samples, 10 g of finely chopped sausage sample was added to 100 mL of distilled water and thoroughly mixed for 15 min. After mixing, the pH value was measured using a pH meter (HANNA HI 9318, Leighton Buzzard, UK).

### 2.7. Determination of Lipid Oxidation (TBARS)

The degree of lipid oxidation was determined using the TBARS test (thiobarbituric acid reactive substances), according to the procedure described by Zhang et al. [28]. Ten grams of finely chopped sausage sample were homogenized with 30 mL of 7.5% trichloroacetic acid (Fisher Scientific, Loughborough, UK) for 2 min at 6000 rpm. The homogenized mixture was filtered, and 5 mL of 20 mmol/L thiobarbituric acid (ISOLAB Laborgeräte GmbH, Wertheim, Germany) was added. This mixture was incubated at 95 °C for 30 min, cooled to room temperature, and the absorbance was measured at 532 nm. A standard curve was prepared using a series of 1,1,3,3-tetraethoxypropane solutions, i.e., malondialdehyde (MDA) (Sigma-Aldrich, St. Louis, MO, USA), by diluting a 10 mM stock solution at a 1:500 ratio in distilled water. All analyses were performed in triplicate, and the results are expressed as mg MDA/kg of meat.

### 2.8. Microbiological Analysis of Sausage Samples

For the microbiological analysis of fresh pork sausages, 25 g of sample was added to 225 mL of sterile peptone water (containing 0.8 g/L NaCl and 1 g/L peptone) and mixed continuously at room temperature for 15 min. Subsequently, serial dilutions were prepared, and a 1 mL aliquot of the appropriate dilution was transferred to a sterile Petri dish, followed by the addition of the corresponding selective nutrient medium. The total viable bacterial count was determined using nutrient agar (Torlak, Belgrade, Serbia); the number of lactic acid bacteria (LAB) was assessed on MRS agar (HiMedia Laboratories, Kampenhout, Belgium); and yeast and moulds were determined on Sabouraud maltose agar (Torlak, Belgrade, Serbia). After incubation at 30 °C for 48 h for bacteria and at 25 °C for 72 h for fungi, colony counting was performed, and the results were expressed as log CFU (colony-forming unit)/g.

### 2.9. Sensory Analysis of Raw and Thermally Processed Sausages

The sensory evaluation of fresh and thermally processed pork sausages was carried out by 10 consumers from the Department of Food Technology at the Faculty of Technology in Leskovac according to the ISO 11136:2014 Sensory analysis—Methodology—General guidance for conducting hedonic tests with consumers in a controlled area [29]. A short training session was conducted prior to the sensory evaluation to ensure a consistent understanding of the assessed attributes and scoring criteria among panellists. The sensory analysis of raw pork sausages included a descriptive analysis of the parameters colour, odour, and overall acceptability. The fresh sausage samples were roasted each day of sampling at 200 °C in a convection oven for 8 min. The sensory analysis of thermally processed sausages involved the evaluation of sensory parameters such as colour, appearance, texture, juiciness, taste, odour, and overall acceptability. Each parameter was rated on a scale from 1 to 5, where a score of 1 represented extremely unacceptable, and a score of 5 represented extremely acceptable characteristics of the analyzed sausage sample. Sensory scores were calculated as the mean of three replicate measurements obtained from independent samples. Sensory scores below 3 were considered below an acceptable level [28]. The consistency of panel evaluations was verified by ensuring that the coefficient of variation for each attribute did not exceed 20%.

### 2.10. Statistical Analysis

All experiments were performed in triplicate, and the results are expressed as mean ± standard deviation. Differences were assessed using one-way ANOVA followed by Tukey’s post hoc test. A *p*-value of less than 0.05 was considered statistically significant. All analyses were carried out with SPSS version 21.0 (IBM, Armonk, NY, USA).

## 3. Results and Discussion

### 3.1. Chemical Composition of Caraway (Carum carvi L.) Essential Oil

**Figure 1:**
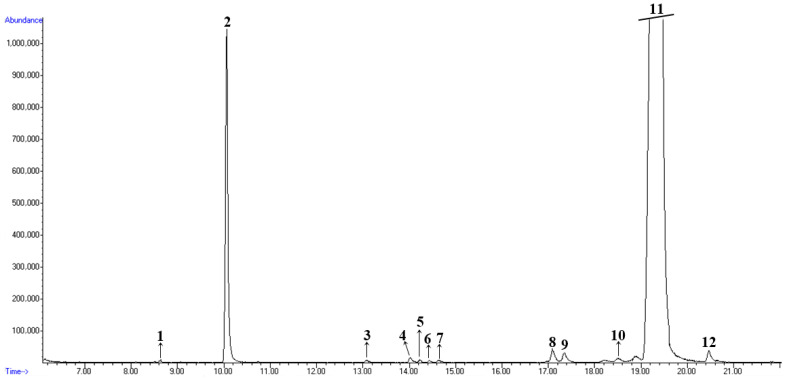
Total ion chromatogram of caraway (*Carum carvi* L.) essential oil. The numbers correspond to the components in the Table 2.

Based on the results obtained from GC/MS and GC/FID analyses, the chemical composition of caraway (*Carum carvi* L.) essential oil represents a complex mixture of monoterpene hydrocarbons and oxygenated monoterpenes(Figure 1). The peak at a retention time of 10 min corresponds to limonene, with a content of 5.80% in the essential oil, while the peak at a retention time of 19.5 min corresponds to carvone, with a content of 92.50%. These two components constitute the highest percentage in the obtained essential oil (Table 2). According to the literature [7], carvone and limonene are primarily responsible for the characteristic aroma of caraway seeds.

The remaining components in CEO are present in significantly smaller amounts, primarily as monoterpene hydrocarbons. The retention index values determined experimentally using a homologous series of n-alkanes are close to the retention index values reported in the literature [26] (Table 2).

In the study by Gajć et al. [8], it was found that limonene and carvone were the dominant components of CEO, constituting 25.6% and 72%, respectively. Similar results were obtained by Aly et al. [30], where the content of limonene was 27.47% and carvone was 56.80% in a CEO sample from Egypt. Other studies show that CEO from Iran primarily contains γ-terpinene (23.78–40.87%) as its main component, and the content of this compound, as well as other components, depends on the flowering period of the plant [31]. In the same study, the content of oxygenated monoterpenes is significantly lower (21.91–42.75%) compared to the present study. The proportion of oxygenated monoterpenes is notably higher in essential oils of caraway obtained from plants cultivated in different regions of Morocco (73.06–81.43%). The type and proportion of components in the chemical composition of CEO depend on the geographical origin, flowering period, cultivation conditions, and seed maturity [32,33], as well as the method of oil extraction [34].

### 3.2. Efficacy of Caraway Essential Oil Encapsulation

The encapsulation efficiency of CEO within chitosan nanoparticles was 67.7 ± 1.91%. This data is consistent with the literature, where the encapsulation efficiency of CEO within nanoemulsions ranged from 46.35% [35] to 78.45% [36]. In contrast, a significantly lower encapsulation efficiency of CEO within a chitosan matrix was reported, at only 27% [37]. Variations in the encapsulation efficiency of essential oils within different systems can be explained by the fact that the efficiency of the process can depend on several factors, such as the type of polymer, its concentration, solubility in water or organic solvents, concentration of the added oil, and others [38].

### 3.3. Changes in pH Values of Sausage Samples During Storage

Changes in pH in meat and meat products are most commonly the result of microbial growth and proliferation. Microbial synthesis of organic acids results in a decrease in pH value. On the other hand, degradation of proteins and breakdown of low-molecular-weight compounds by microbial enzymes can induce an increase in the pH value [39]. The pH value represents a critical parameter that can significantly affect the quality of the final product, as it directly influences colour, tenderness, and water-holding capacity [40]. Changes in pH values of fresh pork sausages during five days of refrigerated storage are presented in Table 3.

The pH fluctuation pattern observed across all samples with an initial rise from day 0 to day 2, a decrease on days 3–4, and a partial recovery on day 5, indicating the biochemical changes during refrigerated storage. The early pH rise is predominantly driven by protein catabolism and accumulation of alkaline compounds such as ammonia, biogenic amines, and volatile basic nitrogen, produced by proteolytic bacteria and endogenous muscle enzymes [39]. As storage progresses, lactic acid bacteria (LAB), present in all samples from day 0 and increasing throughout storage, shift the metabolic balance by fermenting residual sugars and glycogen, generating lactic and acetic acid, affecting the mid-storage pH decrease [40]. Ongoing lipid oxidation, evidenced by progressive MDA accumulation (Table 4), further contributes to the acidic shift due to the generation of short-chain organic acids as secondary oxidation products [4]. The partial pH recovery on day 5 is consistent with renewed dominance of proteolytic activity at higher microbial loads, as alkaline metabolites again accumulate once the buffering capacity of the meat system is exceeded [39,41]. Similar trends have been reported in meat products treated with essential oils, where an increase in pH was observed toward the end of refrigerated storage [17]. Initial pH values of all samples were comparable and ranged between 5.81 and 5.88, with no significant differences observed (*p* > 0.05). On the first day of storage, treated samples generally showed lower pH values compared to the control, although statistically significant differences (*p* < 0.05) were observed only for samples treated with the highest concentration of CEO, both in free form and incorporated into chitosan nanoparticles. On the second day, samples containing CEO, regardless of the application method, exhibited significantly lower pH values (*p* < 0.05) compared to the control and the sample treated only with chitosan nanoparticles, indicating the effect of essential oil concentration rather than the presence of chitosan alone. During mid-storage (third and fourth days), lower pH values were generally observed in samples treated with higher concentrations of CEO, with the most pronounced effect recorded in samples containing chitosan nanoparticles with incorporated essential oil. In particular, samples CNPs CEO 0.4 and CNPs CEO 0.6 exhibited significantly lower pH values compared to the control and other treatments, while differences among samples treated with free essential oil were less pronounced. The lowest pH value during the entire storage period was recorded in the CNPs CEO 0.6 sample on the fourth day (5.66), representing a statistically significant decrease (*p* < 0.05) compared to the initial value. On the final day of storage, the control and CNPs samples exhibited the highest pH values, whereas samples treated with higher concentrations of CEO showed lower pH values. A similar concentration-dependent trend was observed in samples containing nanoparticles with incorporated essential oil. Notably, the CNPs CEO 0.6 sample showed minimal pH fluctuations throughout storage and maintained a final pH value close to the initial measurement, indicating improved pH stability. This suggests that the incorporation of CEO into chitosan nanoparticles enhances its effectiveness in controlling pH changes during storage. This can be explained by chemical and indirect microbiological mechanisms caused by CEO- and CNPs CEO in sausages. The mild acidity of CEO constituents, particularly carvone and limonene, contributes to the slight acidification of the meat matrix. Additionally, the acidity of the chitosan nanoparticle system may further reduce local pH in the aqueous phase of the meat upon mixing [14]. Additionally, CEO and CNPs CEO selectively suppress faster-growing proteolytic and aerobic spoilage bacteria, as evidenced by significantly lower TBC values in treated samples (Table 5). The more pronounced pH reduction in CNPs CEO samples compared with free CEO at equivalent concentrations reflects the sustained release of carvone and limonene from the chitosan matrix, maintaining prolonged antimicrobial pressure against proteolytic bacteria [11,12]. This is consistent with CNPs CEO 0.6 exhibiting the lowest and most stable pH values throughout storage, corresponding to its strongest overall effect. Comparable findings have been reported in meat systems treated with nanoemulsified essential oils, where higher concentrations were required to achieve pH stabilization during refrigerated storage [36]. However, less pronounced effects have been observed in fish products treated with chitosan coatings combined with essential oil nanoemulsions, indicating that the effectiveness of such systems depends on the food matrix [42]. Similar pH-stabilizing trends have been reported in beef treated with chitosan coatings incorporating citrus essential oil, where treated samples maintained significantly lower pH values than untreated controls throughout refrigerated storage [16].

### 3.4. Changes in the Degree of Lipid Oxidation in Sausage Samples

Lipid oxidation represents one of the main factors limiting the quality and acceptability of meat and meat products. Malondialdehyde (MDA), as the primary degradation product of lipid oxidation, is commonly used as an indicator of oxidative stability in meat products [4]. The degree of lipid oxidation in fresh pork sausage samples during refrigerated storage is expressed as mg MDA/kg of fresh sausages and presented in Table 4.

**Table 4:** Malondialdehyde (MDA) content of fresh pork sausages during storage (mg/kg).

	Days of Storage					
Samples	0	1	2	3	4	5
Control	1.41 ± 0.09 c,A	1.34 ± 0.09 c,A	1.57 ± 0.04 b,A	1.68 ± 0.05 b,A	1.71 ± 0.05 b,A	2.18 ± 0.10 a,A
CEO 0.2	1.08 ± 0.04 e,B	0.91 ± 0.01 f,B	1.35 ± 0.02 d,B	1.46 ± 0.02 c,B	1.68 ± 0.02 b,A	1.92 ± 0.01 a,B
CEO 0.4	1.01 ± 0.07 d,B	0.93 ± 0.02 d,B	1.18 ± 0.09 c,C	1.12 ± 0.03 c,D	1.38 ± 0.01 b,B	1.79 ± 0.08 a,C
CEO 0.6	1.07 ± 0.13 d,B	0.84 ± 0.03 e,BC	1.20 ± 0.01 b,C	1.05 ± 0.05 d,D	1.18 ± 0.05 bc,C	1.37 ± 0.07 a,E
CNPs	0.85 ± 0.07 d,C	0.94 ± 0.04 d,B	1.15 ± 0.07 c,C	1.30 ± 0.18 bc,C	1.34 ± 0.09 b,B	1.62 ± 0.05 a,D
CNPs CEO 0.2	0.74 ± 0.05 d,C	0.84 ± 0.03 d,BC	1.01 ± 0.08 c,D	1.12 ± 0.03 c,D	1.30 ± 0.07 b,B	1.59 ± 0.09 a,D
CNPs CEO 0.4	0.82 ± 0.09 c,C	0.76 ± 0.07 c,D	0.96 ± 0.07 b,D	1.02 ± 0.06 b,D	1.15 ± 0.07 a,C	1.25 ± 0.04 a,F
CNPs CEO 0.6	0.77 ± 0.04 c,C	0.74 ± 0.07 c,D	0.94 ± 0.09 b,D	1.08 ± 0.04 a,D	1.17 ± 0.09 a,C	1.15 ± 0.04 a,F

Control—untreated fresh sausages; CEO—fresh sausages enriched with caraway essential oil in different concentrations; CNPs CEO—fresh sausages enriched with different concentrations of chitosan-based nanoparticles loaded with caraway essential oil; CNPs—fresh sausages enriched with chitosan nanoparticles; a–f different small letters indicate significantly different results (*p* < 0.05) in the same row; and A–F different capital letters indicate significantly different results (*p* < 0.05) in the same column.

The lowest MDA values were recorded immediately after sample preparation and on the first day of storage in all analyzed samples. In the majority of samples, MDA concentrations increased progressively during the remaining storage period, indicating ongoing lipid oxidation. On the final, fifth day of storage, a significant increase in MDA values (*p* < 0.05) was observed in the control sample, as well as in samples treated with CEO and in samples containing chitosan nanoparticles without incorporated essential oil (CNPs). Throughout the entire storage period, the control sample exhibited significantly higher (*p* < 0.05) MDA values compared to all treated samples, reaching 2.18 mg MDA/kg on the final day. In contrast, samples treated with chitosan nanoparticles, particularly those incorporating CEO, showed a slower rate of lipid oxidation. Initial MDA values were notably lower in samples containing chitosan nanoparticles, both with and without essential oil, compared to samples treated with CEO, indicating an early protective effect of the nanoparticle system. A concentration-dependent effect was observed in samples treated with CEO, regardless of the application method. As the concentration of essential oil increased, lower MDA values were generally recorded, with the most pronounced antioxidant effect observed in samples containing nanoparticles with incorporated essential oil. In particular, the CNPs CEO 0.4 and CNPs CEO 0.6 samples showed minimal increases in MDA values during the final days of storage, with no statistically significant differences observed between the last storage days. These samples reached final MDA concentrations of approximately 1.20 mg MDA/kg, which was about 50% lower than the control sample. The reduced lipid oxidation observed in samples containing nanoparticles with incorporated CEO can be attributed to the antioxidant activity of the essential oil and its sustained release from the chitosan matrix. The antioxidant potential of CEO is associated with its complex chemical composition (Table 2). Carvone is a biologically active compound with pronounced antioxidant potential [43], while limonene exhibits strong anti-inflammatory and antioxidant effects [44]. Other monoterpene compounds detected in CEO, such as myrcene and linalool [45], also demonstrate significant antioxidant potential. Although these compounds were present in smaller concentrations or traces, they may act synergistically, significantly contributing to the overall antioxidant capacity of the oil [46].

Although no official upper limit for MDA concentration in meat and meat products has been established, values around 2 mg/kg are considered indicative of oxidative deterioration, which is often associated with the development of rancid odour [47]. All treated samples showed better results compared to the control sample in this study; however, the addition of CEO to chitosan-based nanoparticles was more effective in reducing lipid oxidation in fresh pork sausages during the 5-day storage period. A similar antioxidant effect was observed in fresh beef, where the presence of caraway essential oil in nanoemulsion resulted in a halving of MDA concentration (around 1 mg MDA/kg), compared to the control sample (around 2 mg MDA/kg) [18]. A comparable advantage of encapsulated over free cumin essential oil was recently demonstrated by Shahabi et al. [48], who reported significantly lower TBARS values in chilled turkey burgers treated with nano-encapsulated cumin EO compared with free EO at equivalent concentrations, highlighting the consistency of this effect across different Apiaceae-family essential oils and meat matrices. The addition of CEO to chitosan-based nanoemulsion also positively affected the reduction in oxidative changes in sardine fillets [42]. Other studies have shown a less pronounced antioxidant effect of CEO added to nanoemulsion in fresh beef patties, reducing MDA levels by only 0.05 mg MDA/kg compared to the control sample after storage at +4°C for 7 days [36]. CEO at a 1% concentration in fresh beef reduced lipid oxidation, with MDA concentrations after 6 days of storage at 0.47 mg/kg, nearly half the level of the control sample (1.19 mg MDA/kg) [17]. On the other hand, the addition of essential oil to beef sausages did not affect lipid oxidation [49]. The combination of CEO and chitosan reduced lipid oxidation in beef by nearly three times (0.32 mg/kg) compared to the control (1.09 mg/kg) after 6 days of refrigeration [50]

### 3.5. Results of Microbiological Analysis of Sausage Samples

The microbiological quality of fresh pork sausages during refrigerated storage was evaluated by monitoring total bacterial count (TBC), lactic acid bacteria (LAB), and yeasts and moulds (Table 5, Table 6 and Table 7). At the start of the experiment, microbial counts across all sample groups were similar, with 3.34–3.45 log CFU/g for TBC, 3.23–3.31 log CFU/g for LAB, and 1.52–1.78 log CFU/g for yeasts and moulds. As expected for fresh, non-heat-treated meat products stored under refrigeration, microbial counts rose in all samples over the five-day period. The untreated control showed the steepest increase, with TBC, LAB, and yeast and mould counts reaching 6.53 ± 0.01, 6.03 ± 0.02, and 6.12 ± 0.02 log CFU/g, respectively, by day 5.

**Table 5:** Total bacterial count (TBC) during storage of fresh pork sausages (log CFU/g).

	Days of Storage					
Samples	0	1	2	3	4	5
Control	3.35 ± 0.02 f,AB	4.48 ± 0.05 e,A	4.95 ± 0.09 d,A	5.08 ± 0.09 c,A	5.43 ± 0.05 b,A	6.53 ± 0.01 a,A
CEO 0.2	3.45 ± 0.15 f,A	4.35 ± 0.05 e,ABC	4.72 ± 0.01 d,B	4.94 ± 0.07 c,B	5.33 ± 0.01 b,B	6.51 ± 0.01 a,AB
CEO 0.4	3.34 ± 0.02 e,B	4.38 ± 0.17 d,AB	4.57 ± 0.04 c,C	4.64 ± 0.02 c,C	5.30 ± 0.02 b,B	6.23 ± 0.02 a,C
CEO 0.6	3.38 ± 0.01 f,AB	4.21 ± 0.01 e,C	4.35 ± 0.03 d,E	4.55 ± 0.02 c,C	5.07 ± 0.04 b,C	6.03 ± 0.01 a,D
CNPs	3.35 ± 0.02 f,AB	4.36 ± 0.08 e,ABC	4.55 ± 0.01 d,C	4.98 ± 0.04 c,AB	5.29 ± 0.06 b,B	6.46 ± 0.03 a,B
CNPs CEO 0.2	3.35 ± 0.02 f,AB	4.29 ± 0.01 e,BC	4.46 ± 0.02 d,D	4.87 ± 0.01 c,B	5.15 ± 0.13 b,C	6.49 ± 0.02 a,AB
CNPs CEO 0.4	3.35 ± 0.02 f,AB	4.22 ± 0.01 e,C	4.43 ± 0.02 d,D	4.86 ± 0.11 c,B	5.10 ± 0.04 b,C	6.18 ± 0.05 a,C
CNPs CEO 0.6	3.35 ± 0.02 c,AB	4.25 ± 0.11 b,BC	4.20 ± 0.06 b,F	4.27 ± 0.05 b,D	4.28 ± 0.02 b,D	5.94 ± 0.05 a,C

Control—untreated fresh sausages; CEO—fresh sausages enriched with caraway essential oil in different concentrations; CNPs CEO—fresh sausages enriched with different concentrations of chitosan-based nanoparticles loaded with caraway essential oil; CNPs—fresh sausages enriched with chitosan nanoparticles; a–f different small letters indicate significantly different results (*p* < 0.05) in the same row; and A–F different capital letters indicate significantly different results (*p* < 0.05) in the same column.

**Table 6:** Lactic acid bacteria (LAB) count during storage of fresh pork sausages (log CFU/g).

	Days of Storage					
Samples	0	1	2	3	4	5
Control	3.31 ± 0.01 f,A	4.70 ± 0.07 e,A	4.76 ± 0.01 d,A	5.26 ± 0.03 c,A	5.75 ± 0.03 b,A	6.03 ± 0.02 a,A
CEO 0.2	3.28 ± 0.06 f,A	4.52 ± 0.01 e,B	4.68 ± 0.02 d,AB	5.21 ± 0.01 c,AB	5.66 ± 0.03 b,A	5.96 ± 0.03 a,AB
CEO 0.4	3.30 ± 0.02 f,A	4.46 ± 0.02 e,B	4.59 ± 0.03 d,BC	5.16 ± 0.05 c,AB	5.59 ± 0.06 b,A	5.97 ± 0.02 a,AB
CEO 0.6	3.27 ± 0.06 f,A	4.16 ± 0.04 e,D	4.37 ± 0.05 d,D	4.89 ± 0.03 c,C	5.34 ± 0.02 b,B	5.98 ± 0.02 a,AB
CNPs	3.29 ± 0.06 e,A	4.32 ± 0.05 d,C	4.37 ± 0.07 d,D	4.95 ± 0.03 c,C	5.20 ± 0.17 b,BC	5.97 ± 0.01 a,AB
CNPs CEO 0.2	3.29 ± 0.03 e,A	4.11 ± 0.02 d,D	4.52 ± 0.19 c,C	5.12 ± 0.11 b,B	5.23 ± 0.20 b,BC	5.90 ± 0.08 a,B
CNPs CEO 0.4	3.33 ± 0.09 f,A	3.98 ± 0.09 e,E	4.33 ± 0.01 d,D	4.91 ± 0.07 c,C	5.08 ± 0.05 b,C	5.94 ± 0.07 a,AB
CNPs CEO 0.6	3.23 ± 0.03 f,A	3.53 ± 0.01 e,F	3.83 ± 0.05 d,E	4.55 ± 0.01 c,D	5.13 ± 0.02 b,C	5.94 ± 0.06 a,AB

Control—untreated fresh sausages; CEO—fresh sausages enriched with caraway essential oil in different concentrations; CNPs CEO—fresh sausages enriched with different concentrations of chitosan-based nanoparticles loaded with caraway essential oil; CNPs—fresh sausages enriched with chitosan nanoparticles; a–f different small letters indicate significantly different results (*p* < 0.05) in the same row; and A–F different capital letters indicate significantly different results (*p* < 0.05) in the same column.

The addition of CEO to the sausage formulation produced a noticeable, concentration-dependent reduction in microbial growth. Higher concentrations of CEO at 0.6 mg/g showed better results than 0.2 and 0.4 mg/g for all parameters during storage, while all samples with CEO mainly showed a lower number of TBC and yeast and mould counts compared to the control after day 2. On day 1, the CEO 0.6 sample already differed significantly from the untreated group (*p* < 0.05). The bioactive components of caraway seed, carvone and limonene, destabilize bacterial cell membranes, interfering with the cellular life cycle [51], disrupt fungal ergosterol biosynthesis, or alter fungal morphology [52]. The antifungal activity of CEO is broad, with confirmed activity against *Alternaria alternata*, *Aspergillus flavus*, *Aspergillus niger*, *Aspergillus ochraceus*, *Aspergillus parasiticus*, *Fusarium verticillioides*, and *Penicillium digitatum* [24]. In smoked fermented sausages, CEO concentrations of 1.5 and 4.5 μL/mL were found to completely inhibit all analyzed mould species [24]. Comparable antimicrobial benefits of CEO in poultry and other meat products have also been documented [17,19]. At lower concentrations, however, the effectiveness of free CEO was limited due to the hydrophobicity and volatility of essential oil components, which reduce their bioavailability and uniform distribution within a complex meat matrix.

This highlights the use of a chitosan-based nanoparticle system in food preservation. Application of CNPs CEO significantly improved the antimicrobial performance compared to free CEO at the same concentrations. From day 2 onwards, all CNPs CEO samples showed significantly lower bacterial counts than the other treatments (*p* < 0.05), and the inhibitory effect became more pronounced as CEO concentration increased. During days 3 and 4, the CNPs CEO 0.6 sample had TBC values around 4.27 log CFU/g and LAB counts in the range of 3.83–5.13 log CFU/g, significantly lower than all other groups (*p* < 0.05). By day 5, the CNPs CEO 0.6 treatment had the lowest counts across all three parameters: 5.94 ± 0.05 log CFU/g for TBC, 5.94 ± 0.06 log CFU/g for LAB, and 5.03 ± 0.03 log CFU/g for yeasts and moulds. This can be explained by the fact that encapsulation breaks the oil down into much smaller particles with a far greater surface area, distributes it more evenly through the product, and releases it gradually over time rather than all at once [14,53]. This is especially relevant for controlling psychrotrophic species, which continue to grow steadily under refrigeration and require sustained antimicrobial activity to be effectively suppressed [54]. These findings also align well with earlier work showing that incorporating essential oils into nanoparticle-based systems can effectively slow LAB proliferation in fresh meat products [20,21,50].

**Table 7:** Yeast and mould count during storage of fresh pork sausages (log CFU/g).

	Days of Storage					
Samples	0	1	2	3	4	5
Control	1.55 ± 0.06 f,B	3.14 ± 0.02 e,A	4.05 ± 0.05 d,AB	4.86 ± 0.16 c,A	5.95 ± 0.04 b,A	6.12 ± 0.02 a,A
CEO 0.2	1.54 ± 0.02 f,B	3.02 ± 0.03 e,B	3.95 ± 0.04 d,C	4.64 ± 0.02 c,BC	5.85 ± 0.04 b,B	6.07 ± 0.04 a,A
CEO 0.4	1.55 ± 0.04 f,B	2.99 ± 0.03 e,B	3.65 ± 0.01 d,D	4.59 ± 0.02 c,C	5.61 ± 0.04 b,C	5.96 ± 0.07 a,AB
CEO 0.6	1.52 ± 0.01 f,B	2.44 ± 0.02 e,C	3.22 ± 0.10 d,F	3.55 ± 0.03 c,E	5.13 ± 0.01 b,F	5.54 ± 0.01 a,C
CNPs	1.78 ± 0.01 f,A	2.37 ± 0.01 e,D	3.97 ± 0.02 d,BC	4.72 ± 0.01 c,B	5.51 ± 0.04 b,D	5.76 ± 0.23 a,B
CNPs CEO 0.2	1.75 ± 0.23 e,A	2.99 ± 0.03 d,B	4.06 ± 0.07 c,A	4.63 ± 0.03 b,BC	5.42 ± 0.12 a,E	5.51 ± 0.19 a,C
CNPs CEO 0.4	1.66 ± 0.12 f,AB	2.23 ± 0.01 e,E	3.37 ± 0.01 d,E	4.25 ± 0.01 c,D	4.79 ± 0.04 b,G	5.21 ± 0.09 a,D
CNPs CEO 0.6	1.62 ± 0.06 f,AB	1.95 ± 0.01 e,F	2.14 ± 0.01 d,G	3.14 ± 0.02 c,F	4.63 ± 0.01 b,H	5.03 ± 0.03 a,D

Control—untreated fresh sausages; CEO—fresh sausages enriched with caraway essential oil in different concentrations; CNPs CEO—fresh sausages enriched with different concentrations of chitosan-based nanoparticles loaded with caraway essential oil; CNPs—fresh sausages enriched with chitosan nanoparticles; a–f different small letters indicate significantly different results (*p* < 0.05) in the same row; and A–H different capital letters indicate significantly different results (*p* < 0.05) in the same column.

The chitosan itself also contributes directly to the overall antimicrobial effect: its positively charged amino groups interact electrostatically with negatively charged microbial cell surfaces, disrupting membrane integrity [22]. This was confirmed by the CNP treatment, which achieved lower microbial counts than the control across all parameters throughout storage, consistent with prior reports on the standalone antimicrobial potential of chitosan-based systems in fresh meat [15]. At the same time, the CNPs-alone samples were clearly less effective than the CNPs CEO formulations, which confirms that CEO was the primary antimicrobial driver, with chitosan serving as a potentiating delivery system rather than the main active agent. Obtained results are consistent with recent work by Hussain et al. [23], who demonstrated that chitosan-based thyme and oregano essential oil nanoparticles significantly reduced microbial growth and improved oxidative stability in refrigerated meat emulsions, confirming the broader applicability of this preservation approach across different meat systems.

### 3.6. Sensory Analysis of Fresh Sausage Samples

**Figure 2:**
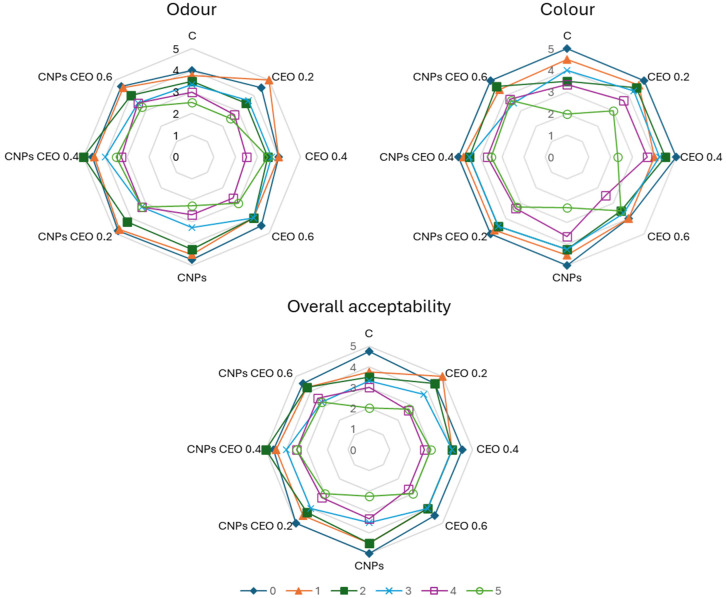
Sensory analysis of fresh pork sausages during 5 days of storage. [◆ 0. day; ▲ 1. day; ◼ 2. day; × 3. day; □ 4. day; and ○ 5. day.] (C—control sample; CEO 0.2—sausages with 0.2 mg/g CEO; CEO 0.4—sausages with 0.4 mg/g CEO; CEO 0.6—sausages with 0.6 mg/g CEO; CNPs—sausages with chitosan nanoparticles; CNPs CEO 0.2—sausages with 0.2 mg/g CNPs with incorporated CEO; CNPs CEO 0.4—sausages with 0.4 mg/g CNPs with incorporated CEO; and CNPs CEO 0.6—sausages with 0.6 mg/g CNPs with incorporated CEO.).

Sensory evaluation of fresh pork sausages indicated high initial acceptability for all samples, with colour, odour, and overall acceptability scores well above the acceptable threshold (score ≥ 3) on the day of preparation (Figure 2). The results of colour evaluation demonstrate that the incorporation of CEO and CNPs CEO significantly influenced the colour characteristics of the samples compared to the control. As storage progressed, a gradual decline in sensory quality was observed in all samples, particularly with respect to odour and overall acceptability. The control sample showed the most pronounced deterioration of sensory attributes, with odour and overall acceptability approaching or falling below the acceptable level toward the end of storage. This decline corresponded with the detected increased lipid oxidation and microbial growth (Table 4, Table 5, Table 6 and Table 7). Also, this can be considered to originate from oxidation of myoglobin to metmyoglobin under refrigerated storage conditions [55]. In contrast, treated samples generally maintained sensory scores above the acceptable limit for a longer period. The incorporation of the CEO contributed to improved odour stability, while nanoparticle-based systems further enhanced this effect, particularly during the later storage days. These results suggest that the monoterpene constituents of CEO, primarily carvacrol and limonene, exert an antioxidant effect that suppresses lipid and pigment oxidation. The CNPs CEO in all concentrations led to the most stable colour scores, suggesting a synergistic protective effect of nanoencapsulation on the bioactive compounds. Encapsulation of essential oils in chitosan-based nanoparticles has been shown to improve the controlled release and efficacy of phenolic antioxidants in meat matrices, thereby better preserving appearance [14].

Odour scores followed a similar trend, with the control sample showing the most rapid deterioration, reflecting the accumulation of volatile off-compounds associated with microbial activity and lipid oxidation. CEO-treated samples retained higher odour scores, particularly at concentrations of 0.4 and 0.6 mg/g, likely attributable to both the pleasant aromatic profile of caraway and the suppression of bacterial activity (Table 5). The deterioration of sensory quality in the control sample, including the development of rancid odour, corresponded well with elevated MDA values (Table 4), confirming the close relationship between lipid oxidation and sensory perception. CNPs alone demonstrated moderate improvements in odour retention compared to the control, consistent with the known antimicrobial properties of chitosan, which disrupt bacterial cell membranes through electrostatic interactions [56]. CNPs CEO samples at 0.2–0.6 mg/g showed odour stability consistently throughout storage, suggesting that nanoencapsulation prolongs the release of aromatic volatiles.

Overall acceptability integrates all perceived sensory attributes and is therefore the most important indicator of product quality. As illustrated in Figure 2, the control sample had the lowest overall acceptability scores, falling below commercially acceptable thresholds by day 5. This pattern is characteristic of fresh sausages without preservative treatments and aligns with the findings of Lorenzo et al. [57], who reported rapid sensory deterioration in minimally processed pork products stored under refrigeration. All treated samples exhibited significantly higher and more sustained overall acceptability scores, with the most pronounced effect observed for CNPs CEO 0.4 and CNPs CEO 0.6. These groups maintained acceptable scores throughout the entire storage period, indicating that the use of CNPs CEO represents a highly effective strategy for extending the sensory shelf life of fresh pork sausages. These sensory findings align with those of Wang et al. [58], who reported that chitosan-based films loaded with lemon essential oil nanoemulsions maintained acceptable colour, odour, and overall sensory quality in chilled fresh pork throughout refrigerated storage, supporting the broader applicability of chitosan–EO nanodelivery systems for fresh pork preservation. According to Dini et al. [18], the addition of cumin essential oil to the nanoemulsion used to treat fresh beef had a positive effect on the colour, odour, texture, and overall acceptability of the meat, maintaining these quality attributes above the acceptable level after 7 days of storage. Similarly, a nanoemulsion incorporating cumin essential oil positively influenced colour retention, preventing discolouration and the development of off-odours and off-flavours in fresh sardine fillets [42].

### 3.7. Sensory Analysis of Thermally Processed Sausage Samples

**Figure 3:**
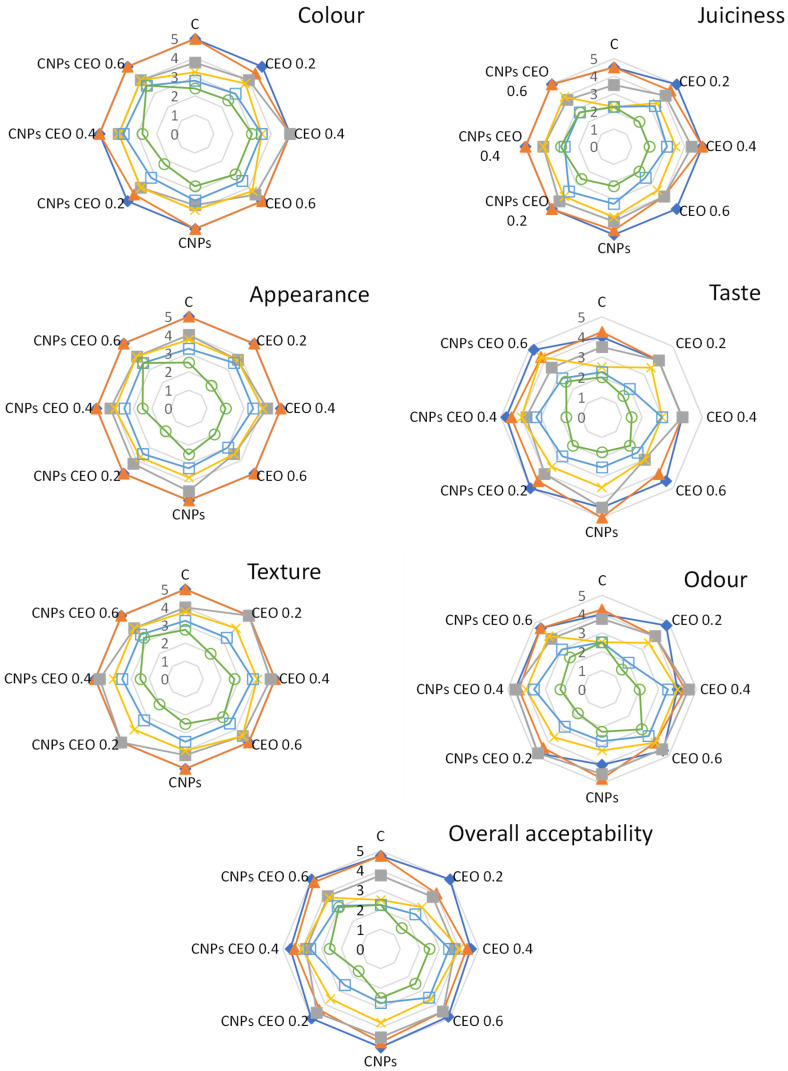
Sensory analysis of thermally processed pork sausages during 5 days of storage. [◆ 0. day; ▲ 1. day; ◼ 2. day; × 3. day; □ 4. day; and ○ 5. day.] (C—control sample; CEO 0.2—sausages with 0.2 mg/g CEO; CEO 0.4—sausages with 0.4 mg/g CEO; CEO 0.6—sausages with 0.6 mg/g CEO; CNPs—sausages with chitosan nanoparticles; CNPs CEO 0.2—sausages with 0.2 mg/g CNPs with incorporated CEO; CNPs CEO 0.4—sausages with 0.4 mg/g CNPs with incorporated CEO; and CNPs CEO 0.6—sausages with 0.6 mg/g CNPs with incorporated CEO.).

The sensory evaluation covered appearance, colour, juiciness, texture, odour, taste, and overall acceptability, with results presented as radar charts (Figure 3).

Thermally processed pork sausages exhibited high sensory quality immediately after preparation, with all evaluated parameters scoring well above the acceptable level. During storage, a gradual decline was observed across all attributes, with significant differences among treatments. The untreated control deteriorated the fastest, particularly in odour and taste, with several attributes approaching or falling below the acceptability threshold by mid- to late storage in correspondence with the reduction in sensory attributes for fresh control sausages.

All treated samples retained higher sensory scores throughout storage, with chitosan nanoparticle-based treatments generally outperforming free CEO. Samples enriched with CEO at 0.4 and 0.6 mg/g maintained a more stable colour and appearance, consistent with findings by Tomović et al. [25], who reported a meaningful positive effect of CEO on colour and odour of dry-fermented sausages. Improved juiciness and texture were especially evident in CNPs CEO-treated samples. Regarding odour and taste, the higher-concentration CEO samples contributed a characteristic spicy, aromatic odour derived from caraway’s dominant volatile compounds D-carvone and limonene (Table 2). Nanoencapsulation allowed the caraway aroma to become more pleasant, with the chitosan nanoparticles moderating the intensity of the sensory effect. This mirrors the experience reported by Sonar et al. [59], who found that chitosan nanoparticles incorporating cinnamon and oregano essential oil delayed off-odour development in thermally treated chicken patties without producing the harsh off-flavours associated with free essential oil application.

## 4. Conclusions

The use of free essential oils in food matrices for prolonged safety has significant limitations, which can be overcome by the application of nanoencapsulated oil. The microbiological data consistently demonstrate that while free CEO provides some antimicrobial benefit, its effectiveness is defined by the physicochemical limitations of direct application in a meat matrix. Encapsulation within chitosan nanoparticles reliably overcomes these limitations and exerts a synergistic effect with chitosan. The CNPs CEO 0.6 formulation is the most effective treatment across all microbiological parameters evaluated, highlighting the potential of this system as a no-additive approach to extending the refrigerated shelf life of fresh pork sausages. All treated samples showed good microbiological and oxidative stability and maintained the bacterial and MDA level below the critical 7 log CFU/g for bacteria and 2 mg MDA/kg for oxidative stability. Considering the sensory analysis, among all treatments, the CNPs CEO 0.6 sample demonstrated the most favourable sensory profile throughout storage, maintaining acceptable scores for all evaluated parameters for the longest period and remaining above the acceptability threshold for odour, taste, juiciness, texture, and overall acceptability of thermally processed sausages. Taken together, these findings confirm that nanoencapsulation of caraway essential oil within chitosan-based nanoparticles contributes to oxidative and microbiological stability and prolongs the shelf life of fresh pork sausages while preserving sensory quality. On the other hand, the storage period of 5 days may not reflect the full potential of CNPs CEO application, so this shortcoming should be addressed in future research. Due to the industrial potential of findings, future research should also address the release kinetics of CEO under simulated gastrointestinal and meat-matrix conditions, evaluating CNPs CEO systems in combination with modified atmosphere or vacuum packaging, and scaling the CNPs CEO preparation method to pilot-plant conditions to evaluate its technological feasibility and economic viability for industrial sausage production.

## Figures and Tables

**Table 1:** Description of the applied treatments to raw pork sausages.

Sample	Description
C	Control sausage sample
CEO 0.2	Sausages with 0.2 mg/g caraway essential oil
CEO 0.4	Sausage sample with 0.4 mg/g caraway essential oil
CEO 0.6	Sausage sample with 0.6 mg/g caraway essential oil
CNPs	Sausage sample with chitosan nanoparticles
CNPs CEO 0.2	Sausage sample with 0.2 mg/g chitosan nanoparticles with incorporated caraway essential oil
CNPs CEO 0.4	Sausage sample with 0.4 mg/g chitosan nanoparticles with incorporated caraway essential oil
CNPs CEO 0.6	Sausage sample with 0.6 mg/g chitosan nanoparticles with incorporated caraway essential oil

**Table 2:** **Results of** chemical composition of caraway (*Carum carvi* L.) essential oil.

No.	t^Ret^, min	Compound	RI^exp^	RI^lit^	Method	Aroma	C, %
1.	8.63	Myrcene	978	988	RI, MS	balsamic, spicy	tr
2.	10.06	Limonene	1020	1024	RI, MS, Co-I	lemon-like	5.8
3.	13.08	Linalool	1100	1095	RI, MS, Co-I	Floral	tr
4.	14.02	trans-p-Mentha-2,8-diene-1-ol	1122	1119	RI, MS	-	0.1
5.	14.23	cis-Limonene oxide	1127	1132	RI, MS	Refreshing	0.1
6.	14.43	trans-Limonene oxide	1132	1137	RI, MS	Refreshing	tr
7.	14.64	cis-p-Mentha-2,8-diene-1-ol	1137	1133	RI, MS	-	tr
8.	17.10	cis-Dihydrocarvone	1196	1191	RI, MS	grass-like	0.4
9.	17.34	trans-Dihydrocarvone	1202	1200	RI, MS	grass-like	0.3
10.	18.52	cis-Carveol	1231	1226	RI, MS	Caraway	0.1
11.	19.42	Carvone	1247	1239	RI, MS, Co-I	Caraway	92.5
12.	20.46	Perillaldehyde	1276	1269	RI, MS	Spicy	0.3
						Total identified (%)	99.6
Distribution by groups (%)						Monoterpene hydrocarbons (1,2)	5.8
						Oxygenated monoterpenes (3–12)	93.8

t^Ret^: retention time; RI^exp^: experimentally determined retention indices (n-alkanes C8–C20, HP-5MS); RI^lit^: literature retention indices [26]; MS: mass spectra; RI: retention index; Co-I: co-injection; tr: trace (<0.05%).

**Table 3:** pH values of fresh pork sausages during storage.

	Days of Storage					
Samples	0	1	2	3	4	5
Control	5.85 ± 0.05 d,A	6.11 ± 0.15 b,A	6.24 ± 0.03 a,A	6.01 ± 0.01 c,AB	6.07 ± 0.04 bc,A	6.19 ± 0.04 a,A
CEO 0.2	5.88 ± 0.07 d,A	6.08 ± 0.03 ab,AB	6.15 ± 0.04 a,B	6.03 ± 0.04 b,A	5.94 ± 0.06 cd,B	6.02 ± 0.03 bc,DE
CEO 0.4	5.83 ± 0.02 e,A	6.04 ± 0.06 b,AB	6.16 ± 0.03 a,B	5.95 ± 0.04 cd,BC	5.91 ± 0.02 d,B	5.98 ± 0.03 bc,E
CEO 0.6	5.81 ± 0.05 d,A	6.01 ± 0.05 b,B	6.12 ± 0.03 a,B	5.91 ± 0.04 c,C	5.89 ± 0.04 c,BC	5.84 ± 0.04 cd,F
CNPs	5.81 ± 0.02 e,A	6.10 ± 0.02 c,A	6.24 ± 0.05 a,A	5.96 ± 0.01 d,ABC	5.82 ± 0.03 e,CD	6.18 ± 0.03 b,AB
CNPs CEO 0.2	5.86 ± 0.04 d,A	6.06 ± 0.07 b,AB	6.17 ± 0.05 a,B	5.94 ± 0.02 c,BC	5.76 ± 0.05 e,DE	6.12 ± 0.04 ab,BC
CNPs CEO 0.4	5.81 ± 0.03 c,A	6.09 ± 0.04 ab,A	6.14 ± 0.02 a,B	5.83 ± 0.04 c,D	5.70 ± 0.02 d,EF	6.06 ± 0.06 b,CD
CNPs CEO 0.6	5.82 ± 0.03 b,A	5.91 ± 0.01 ab,C	6.00 ± 0.03 a,C	5.83 ± 0.09 b,D	5.66 ± 0.09 b,F	5.83 ± 0.04 b,F

Control—untreated fresh sausages; CEO—fresh sausages enriched with caraway essential oil in different concentrations; CNPs CEO—fresh sausages enriched with different concentrations of chitosan-based nanoparticles loaded with caraway essential oil; CNPs—fresh sausages enriched with chitosan nanoparticles; a–e different small letters indicate significantly different results (*p* < 0.05) in the same row; and A–F different capital letters indicate significantly different results (*p* < 0.05) in the same column.

## Data Availability

The original contributions presented in this study are included in the article. Further inquiries can be directed to the corresponding author.
